# Harmonization of the ICHOM Quality Measures to Enable Health Outcomes Measurement in Multimorbid Patients

**DOI:** 10.3389/fdgth.2020.606246

**Published:** 2020-12-15

**Authors:** Mathias C. Blom, Mona Khalid, Britt Van-Lettow, Henk Hutink, Stefan Larsson, Stan Huff, Martin Ingvar

**Affiliations:** ^1^The Boston Consulting Group, Boston, MA, United States; ^2^International Consortium for Health Outcome Measurement, London, United Kingdom; ^3^Nictiz, The Hague, Netherlands; ^4^The Boston Consulting Group, Stockholm, Sweden; ^5^University of Utah Department of Biomedical Informatics, Intermountain Health Care, Salt Lake City, UT, United States; ^6^Department of Clinical Neuroscience, Karolinska Institutet, Solna, Sweden; ^7^Department of Clinical Neuroradiology, Karolinska University Hospital, Solna, Sweden

**Keywords:** health data interoperability, outcomes measurement, standardization, machine learning, patient centered

## Abstract

**Objectives:** To update the sets of patient-centric outcomes measures (“standard-sets”) developed by the not-for-profit organization ICHOM to become more readily applicable in patients with multimorbidity and to facilitate their implementation in health information systems. To that end we set out to (i) harmonize measures previously defined separately for different conditions, (ii) create clinical information models from the measures, and (iii) restructure the annotation to make the sets machine-readable.

**Materials and Methods:** First, we harmonized the semantic meaning of individual measures across all the 28 standard-sets published to date, in a harmonized measure repository. Second, measures corresponding to four conditions (Breast cancer, Cataracts, Inflammatory bowel disease and Heart failure) were expressed as logical models and mapped to reference terminologies in a pilot study.

**Results:** The harmonization of semantic meaning resulted in a consolidation of measures used across the standard-sets by 15%, from 3,178 to 2,712. These were all converted into a machine-readable format. 61% of the measures in the 4 pilot sets were bound to existing concepts in either SNOMED CT or LOINC.

**Discussion:** The harmonization of ICHOM measures across conditions is expected to increase the applicability of ICHOM standard-sets to multi-morbid patients, as well as facilitate their implementation in health information systems.

**Conclusion:** Harmonizing the ICHOM measures and making them machine-readable is expected to expedite the global adoption of systematic and interoperable outcomes measurement. In turn, we hope that the improved transparency on health outcomes that follows will let health systems across the globe learn from each other to the ultimate benefit of patients.

## Background

Value-based healthcare aims to transform care by aligning different stakeholders to focus on patient centric outcomes. Instead of promoting volumes of care services, value-based health care incentivizes value (1–4), with value defined as health outcomes per unit cost for defined conditions over the full cycle of care (1). As the definition of outcomes will impact behavior in value-based contracting, it is paramount that outcomes capture the circumstances most relevant to patients (1). Additionally, the validity of outcomes measurement requires consistent and objective measurement across sites (1, 5–7), which emphasizes the need for high-quality data.

Efforts to systematically collect outcomes measures along the continuum of care are laborious and in some cases constitute questionable use of clinical resources (8–11). Therefore, leveraging routinely collected data for secondary use in quality measurement is growing in popularity (12, 13). Data quality issues (6, 14, 15) and challenges in repurposing data have caused concerns on validity (6, 14) of the wealth of data available in clinical information systems (16). The latter is frequently discussed in relation to electronic health records (EHRs), which many argue are tailored to support billing processes, rather than quality improvement efforts. Another challenge to secondary use of data is fragmentation across organizations and applications with low interoperability (8, 14, 17–19). This could partly be explained by the influence of individual organizations on health data infrastructure decisions, and frequently results in an organization-centric data architecture, aggravated by business models that make it challenging for healthcare organizations to change provider of health information systems (vendor lock-in) (19). The resulting challenges in extracting, sharing and validly analyzing data remain a barrier to standardized and cost-effective quality measurement (7). Such lack of interoperability may also explain why the impact of digital technology on healthcare systems remains equivocal (17, 19–21), despite having been projected to partly “replace the intellectual functions of the physician” 50 years ago (22).

Also in case of sufficient technical- and legal interoperability to allow sharing of data across systems, profound variability in local capture patterns, standards usage and documentation processes render data incomparable due to different structure and meaning (lack of structural- and semantic interoperability) (7, 8, 23). As interoperability standards allow the normalization of data originating from different sources to a common structure and meaning (6, 23, 24), standards have been proposed as a potential solution to the interoperability challenge. However, additional complexity arises as the successful use of interoperability standards requires valid mapping to source data (7), emphasizing the need for interoperability standards with well-defined elements that are easy to implement.

While condition-centric quality registries have promoted quality and procedure development in healthcare to date (25), a mechanism for timely feedback to healthcare processes is required to fully enable a continuously learning healthcare system (26). In contrast to condition-focused quality measures that have been widely implemented to date, measuring patient centric value in healthcare requires the ability to assess patients suffering from multiple conditions (27).

Setting out to achieve the goal of a global, patient-centric health outcomes standard, two of the coauthors (SL, MI) together with Professor Michael Porter from Harvard Business School founded the non-profit international consortium for health outcomes measurement (ICHOM) in 2012 (28). Since its foundation, ICHOM has convened groups of clinical experts and patients from across the globe to define collections of standard outcome measures and risk factors (“standard-sets”) for conditions covering almost 50% of the global disease burden, using a structured consensus process (29). In addition to measures, which are designed to be patient-centric and focus on aspects of health that matter most to the patient, ICHOM standard-sets provide guidance for measurement (e.g., time points, inclusion criteria etc.).

The patient-centeredness and global footprint of the ICHOM standard-sets contrast several other efforts to define quality metrics in healthcare. Committed to ensure sufficient depth of analysis, ICHOM deliberately deploys a condition-centric approach to developing the standard-sets. However, the high level of independence of individual expert groups resulted in insufficient harmonization of measures, and several examples exist where identical measures occur in different standard-sets but are labeled with different unique identifiers, as well as examples of identical unique identifiers mapping to measures of differing meaning. One example of this issue is illustrate by the measure “HORMONTX,” which originally referred to hormonal therapy other than androgen deprivation therapy in the advanced prostate cancer standard-set, but to the intent of hormonal therapy in the breast cancer set. Such ambiguities magnify implementation challenges in health information systems (7) and add to the registration-burden for patients with multimorbidity, who frequently are subject to measurement following multiple standard-sets.

## Objective

Aiming to reduce the burden of implementing ICHOM standard-sets (30), reduce the patient registration-burden and facilitate future alignment with emerging health data standards, the authors set out to harmonize the ICHOM measures and create a repository of measures with well-defined meaning and little redundancy.

## Methods

The harmonization work was executed in two phases, the first of which focused on harmonizing the semantic meaning of measures across all the 28 standard-sets published to date. In the second phase, measures corresponding to four pilot conditions [Breast cancer (31), Cataracts (32), Inflammatory bowel disease (33), and Heart failure (34)] were expressed as logical models and mapped to reference terminologies in a pilot study, which is expected to further facilitate data collection and analysis (35).

### Approaching Harmonization

**Figure 1:**
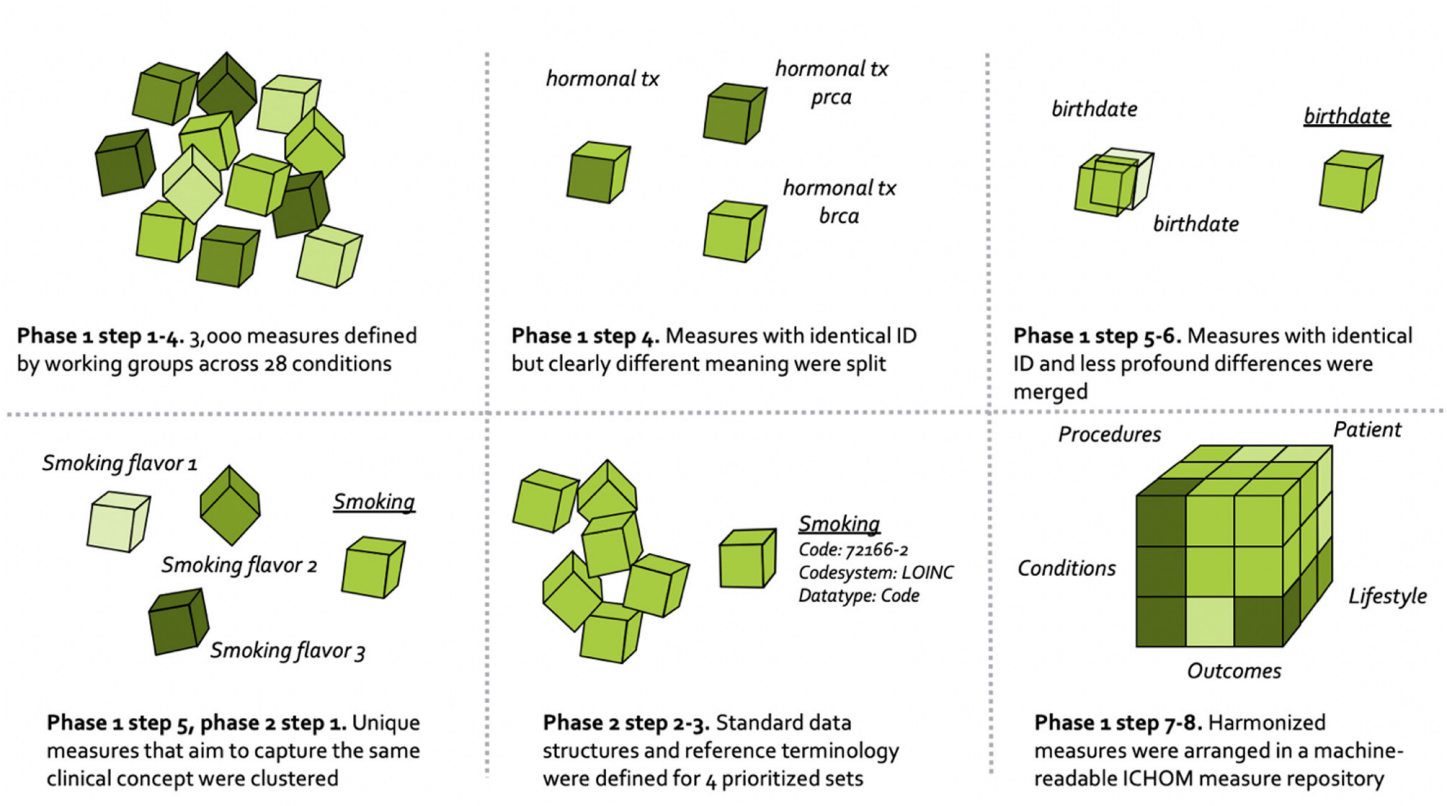
Flow-diagram depicting process for harmonizing the ICHOM measures.

The overall process for harmonizing the ICHOM measures is depicted in Figure 1, and draws on learnings from the field of clinical information modeling (36).

In addition to harmonizing the ICHOM measures across different standard-sets, particular care was taken to specify measure definitions unambiguously, while removing any contextual information specific to individual standard-sets (e.g., regarding timing of measurement, inclusion criteria etc.). Such information is instead expressed in separate execution-logic files (ongoing). This approach of separating the ICHOM information model into two layers aims to increase the recyclability of measures across different conditions and thus facilitate implementation as well as the creation of new standard-sets. Note that measure definitions were iterated as the work progressed, with final definitions agreed on at the end of phase 1 occurring at a point in time after phase 2 was concluded.

### Phase 1–Harmonizing ICHOM Measures Across the 28 Published Sets

The harmonization of ICHOM measures followed the numeric sequence depicted below.

**1. Requirement specification**. Standard-sets of measures covering 28 different conditions were already defined and published at www.ichom.org. These measures have defining attributes depicted below, which are further detailed in the published ICHOM implementation guides (37).

- **Variable ID**. Human-readable unique identifier of the measure.
- **Item**. Human-readable short description of the measure.
- **Definition**. Detailed definition of the measure.
- **Supporting definition**. Further detailed definition of the measure.
- **Inclusion criteria**. Patient population subject to measurement.
- **Timing**. Timing of the measurement.
- **Type**. The required measure data type.
- **Response options**. Response options that are allowed in response to the measure.
- **Reporting source**. The primary source of the information to populate the measure.

**2. Analyzing condition-specific information**. Published ICHOM implementation guides and data dictionaries (all available on www.ichom.org, comprehensive list of conditions available in the Appendix) were studied at the outset of the pilot. Extended search for information was employed to provide additional context where needed.

**3. Indexing the ICHOM measures**. All measures from the 28 published standard-sets were read into a common data structure in the programming language Python (version 3.7.5). After basic formatting, the resulting data dictionary was indexed at the “concept-usage” level (i.e., at the level of the single prescribed ICHOM measure). This was done as to circumvent the indexing issues related to the ambiguous unique identifiers (attribute “Variable ID”) and semantic meanings that were described in the background section. Each row in the resulting dictionary consisted of a unique combination of the attributes “Variable ID,” “Item,” “Definition,” “Supporting definition,” “Response options,” and a unique identifier of each standard-set.

**4. Identifying measures to harmonize**. Differences in semantic meaning of measures across standard-sets were manually assessed by one of the authors (MB). Concept-usages that were judged to be similar enough (different Variable ID with overlapping defining attributes), or different enough (identical Variable ID with differing defining attributes) to be candidates for harmonization were highlighted with a flag and validated by a committee (consensus) consisting of three authors (MB, MK, MI).

**5. Clustering related concept-usages into groups**. Concept-usages assessed as sharing sufficient semantic meaning to warrant consolidation onto a common updated measure (following expert-based method outlined in step 4), were clustered into groups with a human-readable name. All concept-usages from across the 28 conditions (*N* = 3,421) were considered simultaneously, with the concept-usages touching the four pilot conditions subject to an additional step (phase 2 below).

**6. Consolidating related concept-usages onto updated measures**. The concept-usages in each of the related groups generated in step 5 were outputted to spreadsheets and manually consolidated onto single measures defined to cover all concept-usages in a group. In a following step, updated measures were specified using the same defining attributes as described under step 3.

**Figure 2:**
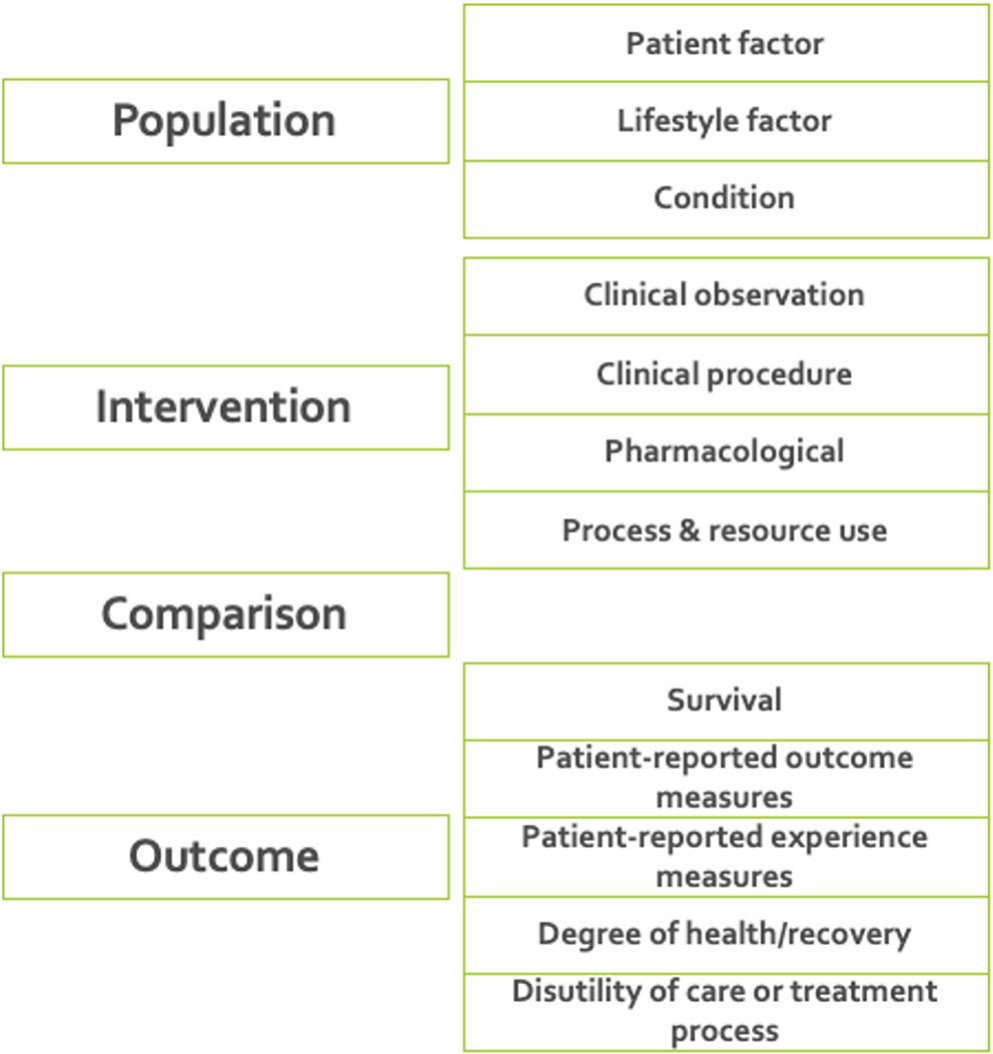
The final taxonomy was organized in two levels, with the first being based on the PICO-model for evidence-based decisions (38). The taxonomy was implemented as a tool for support of further developments including standard-sets for new diagnoses.

**7. Defining ICHOM taxonomy of measures**. A taxonomy was developed for the ICHOM measures, to provide a hierarchical structure and facilitate the identification and retrieval of relevant measures from the ICHOM measure-repository. The final taxonomy was organized in two levels, with the first being based on the PICO-model for evidence-based decisions (38) (Figure 2).

8. **Converting the ICHOM measure-repository to a machine-readable representation**. The measure-repository was converted to JSON format in order to enable reliable versioning. Indexing of response options mapping to each updated measure was also harmonized across sets.

**9. Defining standard-set execution-logic**. A specification of which measures to include for each condition, along with the prescribed timing for measurement, inclusion criteria etc., was defined in a separate resource for each standard-set (ongoing), by transcribing the qualitative information in the implementation guides available at www.ichom.org into a machine readable file following an IF-THEN logic. The specification follows as closely as possible the original recommendations depicted in the implementation guides.

### Phase 2–Defining Logical Models and Terminology

#### Enhancing Related Groups Using Advanced Analytics

To supplement the manual clustering of related concept-usages into groups, phase 2 leveraged advanced analytics to identify additional concept-usages related to those included in the four pilot conditions. A total of 361 concept-usages from the four pilot conditions had been manually clustered into related groups in phase 1 step 4-5. A supervised deep-learning model was then developed to assess concept-usages from across all 28 standard-sets for relatedness to the related groups “touching” the four pilot conditions. The approach included creating semantic embeddings from manually labeled concept-usages (attributes “item,” “definition,” “supporting definition,” and “response options”) that were part of the related groups touching the four pilot conditions, using the FastText algorithm (39). The FastText model is based on the skipgram model, which represents each word as a bag of character n-grams. Words are represented as the sum of these representations, which makes the method fast and allows for computing representations of words not appearing in the training set. In contrast to the traditional skipgram model, FastText deploys a scoring function which takes into account information on the internal structure of words. Context is represented as a bag of the words contained in a fixed size window around the target word. The model deployed in our case was pre-trained on the English Wikipedia using continuous bag of words (CBOW) with position-weights, dimension 300 and 5-length character n-grams. The model was freely available from the FastText GitHub repository (40). Semantic embeddings and their corresponding group labels were fed as inputs to train a supervised Neural Network (MLP Classifier) in Python scikit-learn 0.20.3. A hyperparameter search (three-fold cross-validation, overall accuracy as optimization objective) was conducted before training, to fine-tune model performance. The final model was trained on a random 67% of the observations and evaluated on the remainder 33%. After training, it was subsequently applied to semantic embeddings of all concept usages from across the standard-sets (*N* = 3,421) and used to assign each concept usage a group label from either of the groups touching the pilot conditions. These automatically assigned labels were manually validated by one of the authors (MB). In total, 391 additional concept usages from across all the 28 standard-sets were included in the related groups touching the four pilot conditions, resulting in a total of 752 concept-usages being clustered into these groups. The related groups were converted to XML-format and uploaded to a clinical information modeling platform (41).

#### Clinical Information Modeling

Logical models were defined to cover each group included in the pilot conditions (analogous to phase 1 step 6) and bound to reference terminologies (below). Measures were defined in agreement between three of the authors (MB, MK, MI) and the internal ICHOM standard-set development team, with input from informatics experts at one of the contributing organizations (NICTIZ). The taxonomy-category assigned to a measure guided the modeling approach and the “Response option” attribute guided the specification of value sets to describe which values are allowed in response to each measure (6).

#### Terminology Binding

The measures (including value sets) touching the four pilot conditions were bound to terminology by expert terminologists. Terminologies were deliberately constrained to the international coding systems SNOMED CT (42) and LOINC (43). Measures were defined in a technology-agnostic and human-readable format, and mapped to terminologies only when good matches were available. This design-choice is expected to facilitate re-mappings that may become necessary as technical formats and terminologies evolve (35) as well as promote backwards compatibility with the original versions of the standard-sets.

#### Technical Expert Peer-Review

Upon completion, the draft logical models, value sets and terminology bindings were distributed to individual peer-reviewers in the ICHOM network for review (please see section Acknowledgments). Guidance concerning harmonizing the ICHOM logical models with already available external standards (e.g., FHIR implementation guides), was not always followed, due to a perceived threat to backwards compatibility posed by making ICHOM dependent on independently evolving external standards.

#### Endorsement of Updated Measures by Clinical Expert Groups

After obtaining input from technical peer-reviewers and the chairpersons of the clinical expert groups that developed the original versions of the pilot standard-sets, proposed updates to each of the four sets were circulated to each clinical expert group for review and endorsement. This step is still ongoing for the remaining 24 conditions.

### The ICHOM Measure-Repository

By expressing each measure in a defined syntax, the team aimed to facilitate future creation of computable model serializations (35) (instantiation artifacts) from both measures and execution-logic. While the exact format for these artifacts is not yet agreed, FHIR is a viable candidate (44).

In addition to the attributes defining ICHOM measures prior to harmonization, each measure in the ICHOM measure-repository was enhanced by a set of additional attributes (below) to facilitate versioning and linking to the standard-set specific execution logic files:

- **TERM_ID**. Numeric unique identifier of the measure.
- **Value domains**. Standard data type corresponding to the measure.
- **Concept external code**. Unique code in a reference terminology, corresponding to the measure.
- **Concept external name**. Unique name in a reference terminology, corresponding to the measure.
- **Concept reference terminology**. Reference terminology referred to by attributes “concept external code” and “concept external name”.
- **Displayed value**. Patient-friendly definition of the measure.
- **Taxonomy category**. Category in the ICHOM taxonomy that the measure belongs to.
- **Timestamp**. Timepoint of data recording.

The defining attributes of measures in the measure repository intentionally do not cover information related to the execution of measurement, which is expressed in execution-logic separately defined for each standard-set. The annotation was complemented to improve this. Note that measure definitions may be further modified, as ICHOM expert groups are engaged for feedback.

## Results

The harmonization of the ICHOM measures for the 28 standard-sets resulted in 3,421 original concept-usages (3,178 unique measures) being consolidated onto harmonized measures. In total, 1,081 concept-usages were consolidated into updated measures that replaced two or more of the original concept-usages. The final repository of unique measures contained 2,712 measures, of which 11 were introduced as part of the harmonization process. Of the harmonized measures, 333 touched the four pilot conditions of the second phase. Thus, the creation of the harmonized global ICHOM repository and mappings resulted in a consolidation of the total number of measures by 15%, from 3,178 to 2,712 and all converted to a machine-readable state.

The deep-learning exercise (step 1, phase 2) aiming to improve grouping of the concept usages related to the pilot conditions, resulted in an 84.9% overall accuracy in predicting the correct cluster for a concept-usage, on the test-set.

The clinical information modeling (point 2, phase 2) resulted in 614 unique entities (each analogous to one response option) from across all value sets being linked to the 333 harmonized measures touching the pilot conditions.

Terminology binding (point 3, phase 2) resulted in 399 of the 614 value set items and 182 of the 333 harmonized measures touching the pilot conditions being readily mappable to SNOMED CT or LOINC. This yielded an overall mappable ratio of (399+182)/(614+333) = 61.4%.

Relevant feedback from the expert groups (point 5) was incorporated in the updated measures, again adhering to the ICHOM consensus process, and a total of 9 new measures were introduced by request from the expert groups, across the pilot conditions.

## Discussion

By harmonizing the ICHOM measures, giving them unambiguous definitions and expressing them in a versioned, machine-readable repository we have laid the foundation to expedited integration and implementation of ICHOM health outcomes measurement in clinical information systems. The effects of the harmonization will be particularly notable to patients with multimorbidity and to partners aiming at implementing multiple sets, as harmonizing measures across diagnoses decreases redundancy in measures to be recorded. Harmonization also reduces the risk of conflicting meaning and format of measures, as well as offering new opportunities in risk-adjusting quality measures across diagnoses.

Packaging the proceedings in a versioned, global measure repository also makes it easier to maintain backwards compatibility of measures in the face of the periodic updates that are necessary to keep the standard up to date. Given its associated metadata, human-readable definitions and terminology layer, the measure repository is designed to support the extraction, transform and load (ETL) processes required to enable secondary use of routinely collected data across multiple conditions and applications. Although several initiatives aiming at achieving standardization of meaning in healthcare data exist, we argue that ICHOM is unique in its global and patient-centric approach to defining health outcomes, as well as in prescribing a sequence of measurement required to create transparency on health outcomes.

Although the work reported in this paper aims to make standardized health outcomes measurement easier to implement, additional steps are required to enable a learning healthcare system that would achieve the systematic feedback-loops that start to emerge in the context of other industries, e.g., industry 4.0 (45). One such step is the comparison of performance across providers with subsequent sharing of identified best practices (5), collectively referred to as benchmarking. In turn, the benchmarking process has potential to fuel a virtuous cycle in which organizational learning precipitates repeated performance improvements and innovations. For this to work, the benchmarking process must be connected to an ability to apply learnings in the organization (26, 46). The implementation of benchmarking in healthcare typically requires careful consideration of processes for data collection, as well as the importance of selecting appropriate reference points (benchmarks) (46–49).

Benchmarking has traditionally been considered a management tool, but the importance of engaging clinical staff is increasingly recognized as key to understand practice (46), sources of variation and to create change (26, 27).

It is primarily the desire to involve clinicians that made us opt to maintain definitions sparse and human readable, despite that decomposition of complex ICHOM measures into simpler constituents could result in higher mapping-rates to reference terminologies. The project Steering Committee also decided to limit such post-coordination (23) to prevent overly relying on external terminologies that would make the standard susceptible to semantic drift as terminologies evolve (7). This rationale is also a leading cause of why we have not opted to construct exhaustive clinical information models to cover informational needs outside what was already specified in the original standard-sets.

While the reported work aims to improve the quality of the data collection process, it does not fully address standardized data analysis and benchmarking at scale. In response to this need, work is underway to develop a common data model (CDM) to support distributed analytics, which will be reported separately.

## Conclusion

Standardized health outcomes measurement and the interoperability it brings are key enablers of a patient-centric, learning healthcare system that operates across organizational- and specialty boundaries. By harmonizing the ICHOM measures across conditions and making them machine-readable, we aim to create part of the foundation necessary to enable large-scale collection of high-quality data suitable for quality benchmarking, that can bring about this change.

## Data Availability Statement

The datasets presented in this study can be found in online repositories. The names of the repository/repositories and accession number(s) can be found below: www.ichom.org.

## Author Contributions

MB, SL, SH, MI: concept. MB, BV-L, HH, MI: execution. MB, MI: initial writing. All authors: have contributed to the design and assessment of results of the study as well as read and approved the manuscript.

## Conflict of Interest

MI and SL are co-founders and members of the board of ICHOM and MK was an employee of ICHOM during the completion of this work. The remaining authors declare that the research was conducted in the absence of any commercial or financial relationships that could be construed as a potential conflict of interest.

## Appendix

ICHOM original standard sets subject to harmonization (all available at www.ichom.org)

- Atrial Fibrillation
- Diabetes
- Paediatric Facial Palsy
- Congenital Upper Limb Anomalies
- Inflammatory Arthritis
- Hypertension LMIC
- Chronic Kidney Disease
- Pregnancy Childbirth
- Overactive Bladder
- Colorectal Cancer
- Older Person
- Cranofacial Microsomia
- Dementia
- Coronary Artery Disease
- Localized Prostate Cancer
- Lower Back Pain
- Parkinson Disease
- Depression Anxiety
- Advanced Prostate Cancer
- Cleft Lip Palate
- Lung Cancer
- Hip Knee Osteoarthritis
- Stroke
- Macular Degeneration
- Cataracts
- HeartFailure
- BreastCancer
- Inflammatory Bowel Disease
